# Topological Feature Extraction from Multi-color Channels for Pattern Recognition: An Application to Fundus Image Analysis

**DOI:** 10.1007/s10278-025-01791-1

**Published:** 2025-12-22

**Authors:** Fatih Gelir, Taymaz Akan, Owen T. Carmichael, Md. Shenuarin Bhuiyan, Steven A. Conrad, John A. Vanchiere, Christopher G. Kevil, Mohammad Alfrad Nobel Bhuiyan

**Affiliations:** 1 Division of Clinical Informatics, Department of Internal Medicine, Louisiana State University Health Sciences Center at Shreveport, Shreveport, LA 71103 USA; 2 Pennington Biomedical Research Center, Louisiana State University, Baton Rouge, LA USA; 3 Department of Pathology and Translational Pathobiology, Louisiana State University Health Sciences Center at Shreveport, Shreveport, LA USA; 4 Department of Pediatrics, Louisiana State University Health Sciences Center at Shreveport, Shreveport, LA USA

**Keywords:** Topological feature, Persistent homology, Pattern recognition, Convolutional neural network, Deep learning

## Abstract

The automated analysis of medical images is crucial for early disease detection. In recent years, deep learning has become popular for medical image analysis. In this study, we employed color-based topological features with deep learning for pattern recognition. The data topology provides information about the image’s shape and global features such as connectivity and holes. We used different color channels to identify changes in topological footprints by altering the image’s color. We extracted topological, local binary pattern (LBP), and Gabor features and used machine learning and deep learning models for disease classification. The model’s performance was tested using three open-source fundus image databases: the Asia Pacific Tele-ophthalmology Society (APTOS 2019) data, the Optic Retinal Image Database for Glaucoma Analysis (ORIGA), and the Automatic Detection Challenge on Age-Related Macular Degeneration (ICHALLENGE-AMD). We have found that topological features from different color models provide important information for disease diagnosis.

## Introduction

According to the World Health Organization, over 400 million individuals worldwide suffer from eye diseases such as glaucoma, age-related macular degeneration, or diabetic retinopathy [1]. Many patients are not aware of the progression of their diseases. That is why early detection and treatment are critical. However, the current process of diagnosing these conditions is slow. It requires a specialist to manually analyze retinal images, which can delay treatment, underscoring the ongoing need for clinical decision-support tools.

In recent years, topological data analysis (TDA) and deep learning have demonstrated significant success in medical image analysis, improving diagnostic accuracy and extracting meaningful features from complex data [1–7]. TDA excels at uncovering hidden topological patterns in images and transforming them into meaningful features, providing insights into the underlying structure of complex data. Meanwhile, convolutional neural networks (CNN) are highly effective at learning and identifying intricate patterns directly from the data, making them powerful tools for feature extraction and classification [8, 9]. Together, these approaches offer complementary strengths in analyzing medical images. Using a method from TDA called persistent homology (PH), we can convert these patterns into meaningful image features that classifier models can effectively leverage for accurate classification. Using topological and neural network-based features enables models to leverage both the global topological structure and the local pixel-level information in images, providing a more comprehensive approach to classification tasks.

TDA has been successfully applied across several medical fields for decades, but its usage has been somewhat limited compared to more conventional data analysis techniques. In cancer research [10], TDA has been employed to summarize the geometric information in tumor cell arrangements. Another application of TDA is in fundus image quality assessment [11], where TDA has been used to evaluate the structure and quality of retinal images, assisting in the classification of image quality, which is critical for accurate retinal disease diagnosis. In skin disease image analysis [12], TDA has been applied to classify skin lesions, distinguishing between benign and malignant conditions by analyzing the topological features of skin disease images. Additionally, in pathological image analysis [13], TDA has been used to extract topological features from histopathological images, thereby helping identify disease patterns and support diagnosis across various tissue types.

Furthermore, TDA has been utilized in retinal image screening [14], enabling the detection and classification of retinal abnormalities and thereby facilitating the early diagnosis of conditions such as diabetic retinopathy and glaucoma. In a separate study, topological features derived from fundus images were extracted for the classification of retinal diseases utilizing four color channels [15]. This study focuses on retinal image classification using both machine learning and deep learning frameworks. We first extracted topological features from six color channels—red, green, blue, hue, saturation, and value—to capture structural and geometric properties across multiple color spaces. In addition, local binary patterns (LBP) and Gabor features were derived to represent texture and spatial-frequency information. Based on these features, we applied two complementary models. The first model, Topo-LBP-G-ML, combines topological, LBP, and Gabor features and employs traditional machine learning algorithms for disease classification. The second model, Topo-RetNet, uses the same handcrafted features but integrates them with a deep learning architecture (ResNet50) to capture high-level semantic representations.

## Topological Data Analysis

In this paper, we have applied PH as an effective feature extraction method for images. PH is a fundamental method in TDA, allowing us to analyze the hidden patterns in the data by adjusting a scale parameter [16]. We used cubical persistence [17], a form of PH designed for image data. It analyzes topological features—such as connected components, holes, and cavities—in images by working with cubical complexes, which naturally fit the grid structure of image pixels or voxels.

PH can be broken down into three main steps. The first step is the filtration. It helps to build a bridge between data and its topological features. It also provides information about the data structure. Later, to capture topological features in a dataset, PH is converted to persistence diagrams. These diagrams distinguish between stable and significant features by plotting each topological feature as a point, with its birth (when it appears) and death (when it disappears) on the axes. The distance of points from the diagonal in a persistence diagram, where birth equals death, quantifies the features’ persistence and stability.

Vectorization is the final step that converts these data from persistence diagrams into vector formats usable by conventional ML algorithms [18, 19].

**Fig. 1:**
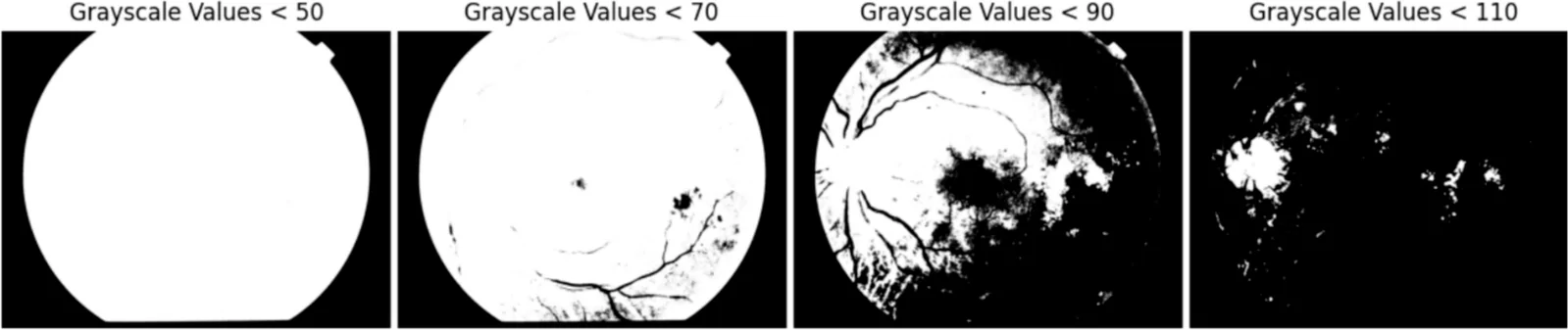
Binary images ${{\boldsymbol{I}}}_{50\boldsymbol{ }},{\mathrm{I}}_{70}$, ${\mathrm{I}}_{90}$, and ${\mathrm{I}}_{110}$ were generated from a fundus image by applying threshold values of 50, 70, 90, and 110

In the context of images, constructing filtrations is different due to the distinct structure of image data. Image filtration involves a series of nested binary images known as cubical complexes. Given a color channel or a grayscale image ${\boldsymbol{I}}$ with dimensions m×n, we first select a color channel (e.g., red, green, blue, or grayscale). The pixel values ${\beta }_{ij}\in \left[0, 255\right]$ represent the color intensities for individual pixels $\delta_{ij}\subset I$, where $\delta_{ij}$ is closed square (boundaries included) at the ${i}_{th}$ row and ${j}_{th}$ column of the image ${\boldsymbol{I}}$. Next, we determine the number of thresholds, denoted as l to span the color range $\left[0, 255\right],$ with $0= {\mathrm{p}}_{1 }< {p}_{2} < \dots < {\mathrm{p}}_{l }=255$. This defines the length of our filtration sequence, usually set between 50 and 100. Using these thresholds, we create a sequence of nested binary images (cubical complexes) ${\mathrm{I}}_{1 }\subset {\mathrm{I}}_{2 } \subset \dots \subset {\mathrm{I}}_{l}$ where ${\mathrm{I}}_{t}$ is defined as $I_n=\{\delta_{ij}\subset I\mid\beta_{ij}\leq p_t\}$ (as illustrated in Fig. 1). This means starting with an empty m×n image and progressively turning pixels black as their grayscale values reach each threshold ${\mathrm{p}}_{t}$. This approach is called sublevel filtration. It is applied to ${\boldsymbol{I}}$ with respect to the chosen color channel (e.g., grayscale). Alternatively, one can activate pixels in descending order of their values, known as superlevel filtration. In this case, let $I_t=\left\{\delta_{ij}\subset I\mid\beta_{ij}\geq q_t\right\}$ where $255= {\mathrm{q}}_{1 }> {\mathrm{q}}_{2 }> \dots> {\mathrm{q}}_{l }=0$, forming the nested sequence${\mathrm{I}}_{1 }\subset {\mathrm{I}}_{2 } \subset \dots \subset {\mathrm{I}}_{l}$, referred to as superlevel filtration [20].

The selection of thresholds is critical in this process. It determines the details the PH will use to identify topological features. One may select N=255, which would result in an overly fine filtration. It will cause most outputs to be trivial during the fingerprinting process. On the other hand, choosing a very small N could lead to numerous topological features being missing. In our study, we chose N=100, which provided the optimal output, and further increasing N did not yield any significant performance improvement.

Persistence diagrams (PDs) are used in the context of image analysis to locate and identify topological structures, such as connected components (0- dimentional features), and loops (1- dimentional features), across various grayscale thresholds. One can observe how these features are born and die at various levels by filtering the pixel values from low to high intensity. For instance, when analyzing a small k×k pixel grayscale image, a filtration process is applied to fill the pixels based on their intensity incrementally. Then, the connected components formed at each step are recorded. PDs provide a visual representation of the features in the image as points with birth and death coordinates corresponding to the grayscale thresholds at which they appear and merge with other features, respectively. In other words, if a topological feature σ appears for the first time at ${\alpha }_{birth} (\sigma )$, and disappears at ${\alpha }_{death} (\sigma )$ the PD is calculated per: ${PD}_{k}\left(I\right)=\left\{{(\alpha }_{birth} \left(\sigma \right), {\alpha }_{death} \left(\sigma \right)\right) \mid {H}_{k}\left({I}_{t}\right) for {\alpha }_{birth} \left(\sigma \right)\le \mathrm{t}< {\alpha }_{death} \left(\sigma \right)\} .$ Here, ${H}_{k}\left({I}_{t}\right)$ indicates ${k}^{th}$ homology group of ${Y}_{t}$, representing k- dimensional topological features in cubical complex ${I}_{t}$. In our case of 2D image analysis, we only use k=0,1, i.e., ${PD}_{0}\left(I\right) \,and\, {PD}_{1}\left(I\right).$ These diagrams help in identifying significant structures in images. More persistent features (with longer lifetimes) typically correspond to important structures.

**Fig. 2:**
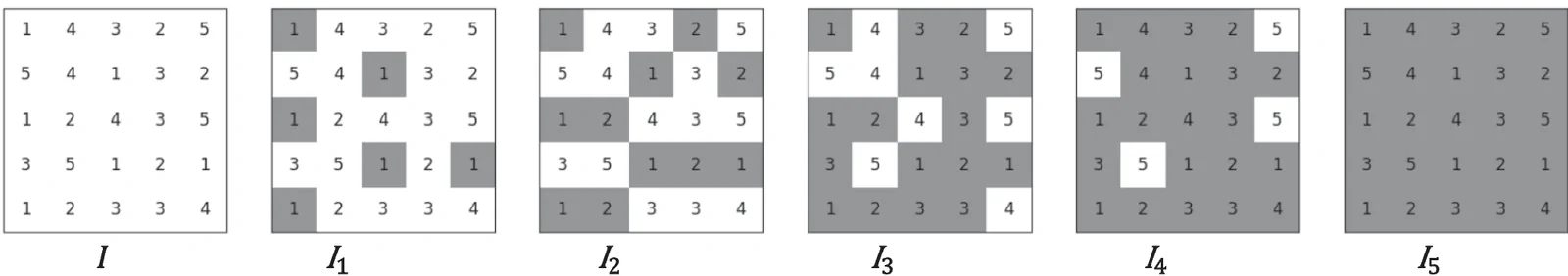
The sublevel filtration is illustrated using a 5×5 image with assigned pixel values. This process generates a series of binary images, where each successive image is a subset of the next, forming a nested structure ${\mathrm{I}}_{1 }\subset {\mathrm{I}}_{2 } \subset \dots \subset {\mathrm{I}}_{5}$. Each image in the sequence represents a sublevel set, progressively incorporating pixels based on their intensity values

Figure 2 shows that there are six components at ${\mathrm{I}}_{1}$, which correspond to six 0- dimensional features with a birth time of 1. At ${\mathrm{I}}_{2}$, the number of components decreases to two. Thus, five 0- dimensional topological features disappear at p=2. Moving to ${\mathrm{I}}_{3}$, there is just one component left, which gives the 0- dimensional PD, ${PD}_{0}\left(I\right)=\{(1,{ \infty }), (1, 2), (1, 2), (1, 2), (1, 2), (1, 4)\}$. For the 1- dimensional features, there are no holes present in ${\mathrm{I}}_{1}$ and ${\mathrm{I}}_{2}$, but two holes emerge at ${\mathrm{I}}_{3}$, marking the birth of two 1- dimensional features with birth time 3. One of these holes dies at ${\mathrm{I}}_{4}$, while another new hole dies at ${\mathrm{I}}_{5}$. Resulting in the 1- dimensional persistence diagram ${PD}_{1}\left(I\right)=\{(3, 4), (3, 5)\}$. PDs, represented as collections of 2-tuples, are not directly suitable for use in machine learning models. To address this challenge, vectorization [21] is commonly employed as the final stage of the persistent homology (PH) process. This technique transforms PDs into vectors or functions, making them more practical for integration into machine learning workflows. Betti function monitors the count of active topological features at a given threshold. this is a step function, where ${\beta }_{0}({ p}_{l})$ indicates the number of connected features in the binary image ${\mathrm{I}}_{l}$. In addition, ${\beta }_{1}({ p}_{l})$ indicates the number of holes or loops. In ML applications, Betti functions then converted into vectors, where each vector ${\overrightarrow{\beta }}_{k}$ has L entries, representing the values of and ${\beta }_{k}({ p}_{l})$ for $1 \le l \le L$. Therefore, we have ${\overrightarrow{\beta }}_{k}\left({\mathrm{I}}_{ }\right)=[ { \beta }_{k}\left({ p}_{1}\right){ \beta }_{k}\left({ p}_{2}\right)\dots { \beta }_{k}({ p}_{L})]$. For instance, in Fig. 2 for the image Y, the vector for ${\beta }_{0}(\mathrm{I})$ is [6,2,2,1,1], meaning that ${\beta }_{0}(1) = 6$ represents the number of connected components in ${\mathrm{Y}}_{1}$, while the vector for ${\beta }_{1}(\mathrm{I})$ is [0,0,2,1,0], and the number of holes or loops in ${\mathrm{I}}_{3}$ were expressed by ${\beta }_{1}(3) = 2$.

### Topological Feature Extraction with Different Color Channels

#### Topological Features from Different Color Models

**Fig. 3:**
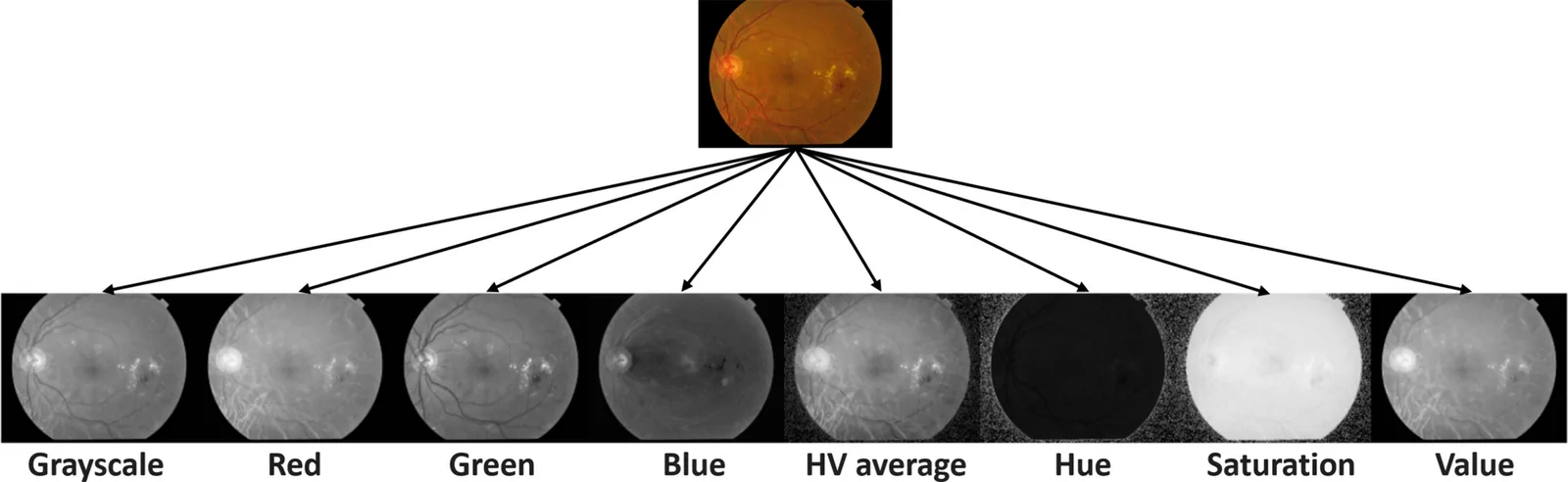
Feature extraction using RGB and HSV (hue, saturation, and value) color channels

**Fig. 4:**
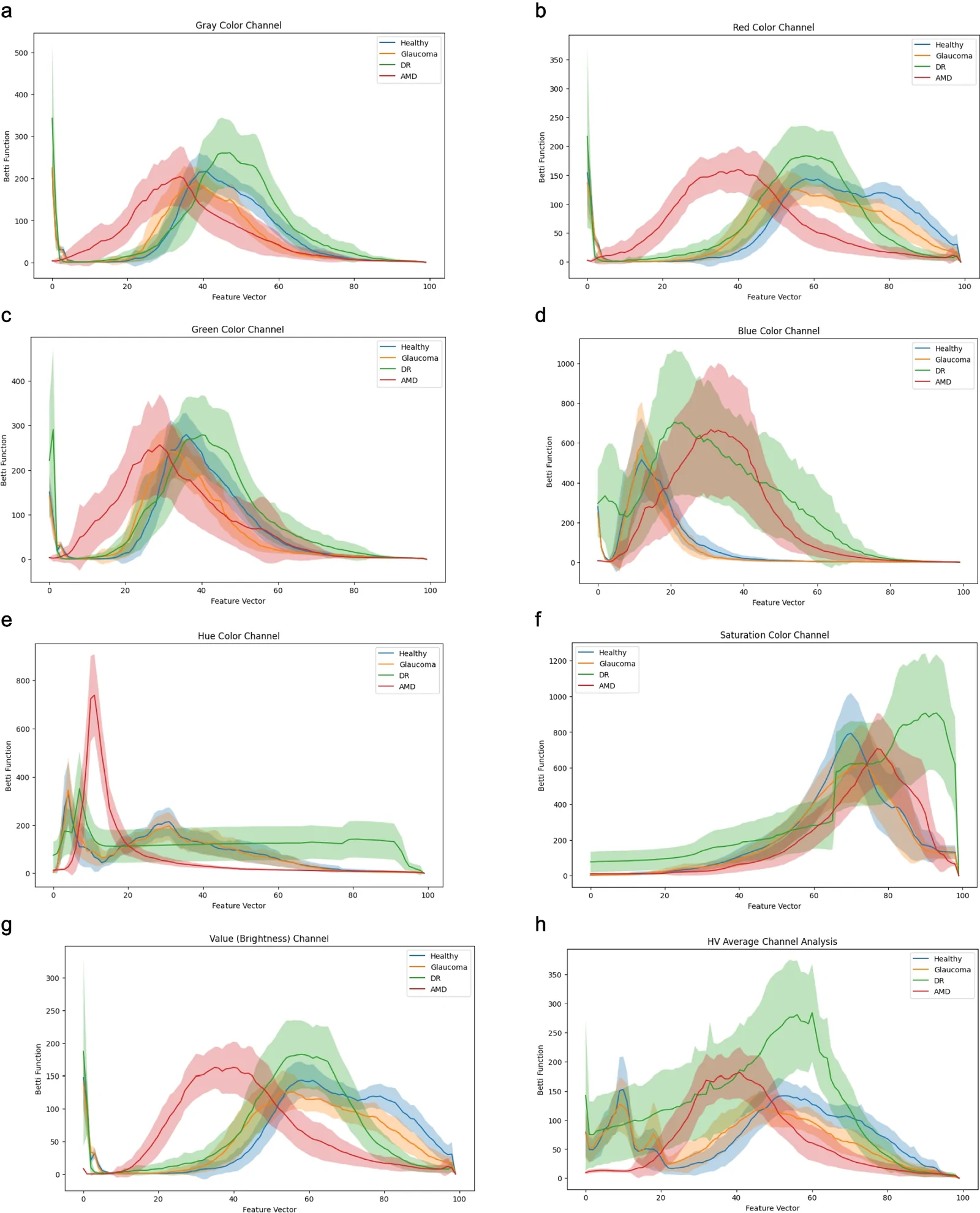
Comparative analysis of Betti-1 curves across different color channels obtained from topological data analysis of retinal images. Panels correspond to: (**a**) grayscale channel, (**b**) red channel, (**c**) green channel, (**d**) blue channel, (**e**) hue channel, (**f**) saturation channel, (**g**) value (brightness) channel, and (**h**) HV-average channel. In each panel, the mean Betti-1 curve is shown for Healthy, Glaucoma, DR, and AMD classes, with shaded regions indicating variability across samples. The plots illustrate how loop-related topological structures vary across color representations and disease categories

Derived features from each retinal image using eight distinct colors. These include the primary red, green, and blue color channels called RGB. Additionally, we incorporate the Grayscale color, computed as a weighted sum of the RGB values, to capture intensity-based information. They applied this approach for histopathological cancer detection, demonstrating the effectiveness of color-based topological features in capturing morphological variations. To enhance feature extraction, we also utilize Hue, Saturation, and Value colors (HSV), to incorporate a perceptually meaningful approach to color analysis. Lastly, we include the mean HSV color, calculated as the mean of the H, S, and V values for each pixel. For a given image I with a resolution p×q, topological feature vectors were created by first extracting values from these eight color channels (RGB, HSV, gray scale and mean HSV) (see Fig. 3). These colors were defined with a function (x,y) which maps each pixel ${\Delta }_{xy }\subset I$ to its associated colors, where $1\le x\le p$ and $1\le y\le q$. By using these eight color functions ((h(x,y)), a sublevel filtration was constructed for each channel and calculated the corresponding persistence diagrams ${PD}_{k}\left(I, h\right)$. To effectively integrate the extracted topological patterns, PDs were converted into Betti functions. We normalized the color values ranging from $[\mathrm{0,255}]$ to $[\mathrm{1,100}]$ with the number of thresholds set to N=100. As a result, each $$\beta \begin{array}{c}h\\ n\end{array}(I)$$ represented as a vector with a dimension of 100. As a result, the eight color models, each with two dimensions (*n* = 0,1), generate 16 unique vectors each with a dimention of 100. In total, for each image I, we extract 1,600 topological features. In Fig. 4, we present the Betti-1 curves of retinal images across eight different color channels.

Next, to capture the local texture pattern from different color channels, we used the LBP extraction [22]. LBP captures the local texture pattern by incorporating the spatial structure of the pixel of an image. To capture the mathematically, for a pixel *I* at coordinates (x, y) the LBP can be calculated as,

1 $$LB{P}_{Q}, f(x, y)=\sum\limits_{q=1}^{Q-1}\left(\text{h }({\mathrm{I}}_{\mathrm{q}}+{I}_{c}\right) . {2}^{q}$$

where ${\mathrm{I}}_{\mathrm{q}}$ represents the intensity of the neighboring pixels, Q is the number of neighboring pixels, f is the radius of the neighborhood, and h(x) is the threshold function, s(x) = 1 if x > 0 or 0 otherwise. Thus, LBP compares the intensity of each neighboring pixel with the center pixel, and If a neighbor’s intensity is greater than or equal to the center pixel, assign it a value of 1; otherwise, assign it 0. LBP features were extracted using a histogram method with a bin size one hundred for each color channel, producing 800 features per image.

Additionally, for spatial frequency, orientation, and edge structures, they used a linear texture analysis tool developed by Dennis Gabor called the Gabor filter [23]. A Gabor filter was used to detect frequency content in localized regions of the image. By adjusting the orientation (θ) and wavelength (ρ), they extracted features from each color channel to enhance pattern recognition. Mathematically the Gabor function can be written as,

2 $$\mathrm{h}\left(\mathrm{m},\mathrm{n}\right)=\mathrm{exp}\left(- \frac{{m}^{2}+ {\beta }^{2}{n}^{2}}{2{\eta }^{2}}\right).\mathrm{cos}\,2\pi \frac{m}{\rho }+ \lambda$$

where m = mcosθ + n sinθ; *n* =—msinθ + n cosθ; *ρ* = Wavelength of the sinusoidal wave; θ = Orientation of the filter in radians; λ= Phase offset of the sinusoidal wave; η = Standard deviation and β = Aspect ratio, determining the shape of the Gaussian. By adjusting the orientation (θ) and wavelength (ρ) of the function, they extracted fifty features from each color channel to enhance pattern recognition. Gabor features were extracted using five different angles $(0,\theta ,\theta /2,\theta /\mathrm{4,3}\theta /4)$ for both θ and ρ values. Using this process, they generated 50 features per color channel (25 local and 25 mean amplitude-based features), resulting 400 features per image.

**Fig. 5:**
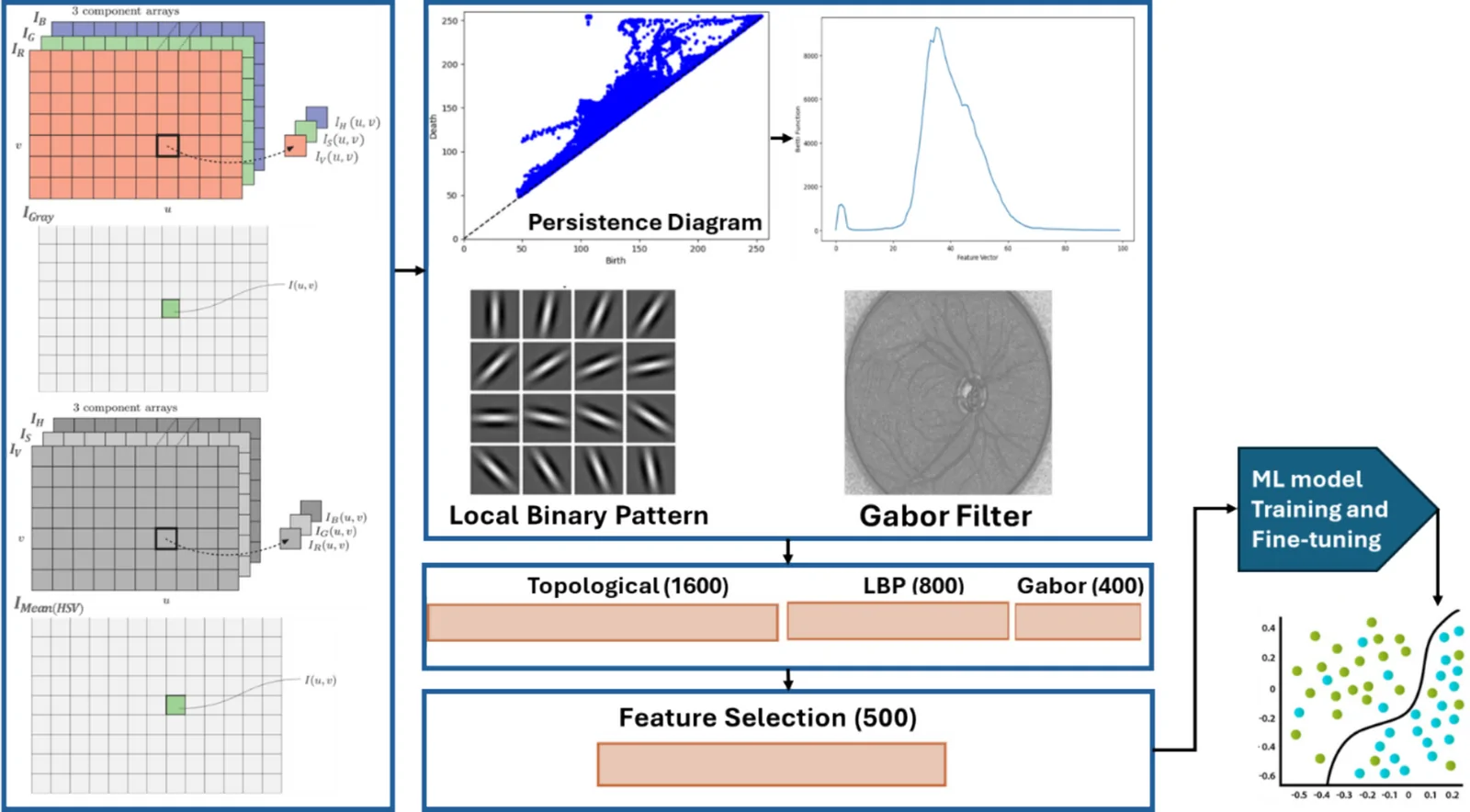
Flowchart of Topo-LBP-G-ML model: The process begins by generating persistence diagrams from the grayscale values of a given retinal image. Then, topological feature vectors were extracted from the diagram using Betti functions. These Betti functions were then used in our machine learning models for classification

We have also applied the same architecture for retinal image analysis. Each retinal image generated 2800 features, comprising 1,600 topological features, 400 Gabor features, and 800 LBP features. This comprehensive feature-extraction process yielded a 2800-dimensional latent-space representation for each image. With the 2800 extracted features per fundus image, we used Extreme Gradient Boosting (XGBoost) [24] for pattern recognition. To avoid overfitting, we used Shapley Additive Explanations (SHAP) [25] to rank features by importance and select them for classification. We focused on the most important features, improving the model’s efficiency and ensuring it performed well without compromising accuracy or interpretability (see Fig. 5 for model architecture).

Pre-trained CNNs and fine-tuning strategies have proven effective for extracting meaningful, high-level representations from medical images. Integrating these features with topological features has shown promise for improving model interpretability and classification accuracy. [26] proposed such an integration model, combining a pre-trained deep network with topological features to capture both geometric and structural characteristics of image data.

In this study, we follow the same architectural framework as [15] and employ ResNet50, a deep learning model pre-trained on the ImageNet dataset. As the CNN backbone for feature extraction. However, unlike [15], that utilized only deep and topological features, our proposed Topo-RetNet model extends this integration by incorporating additional handcrafted descriptors, LBP and Gabor features. These features capture fine-grained texture and spatial-frequency information, complementing deep and topological representations and enabling a more robust, discriminative analysis of retinal images.

**Fig. 6:**
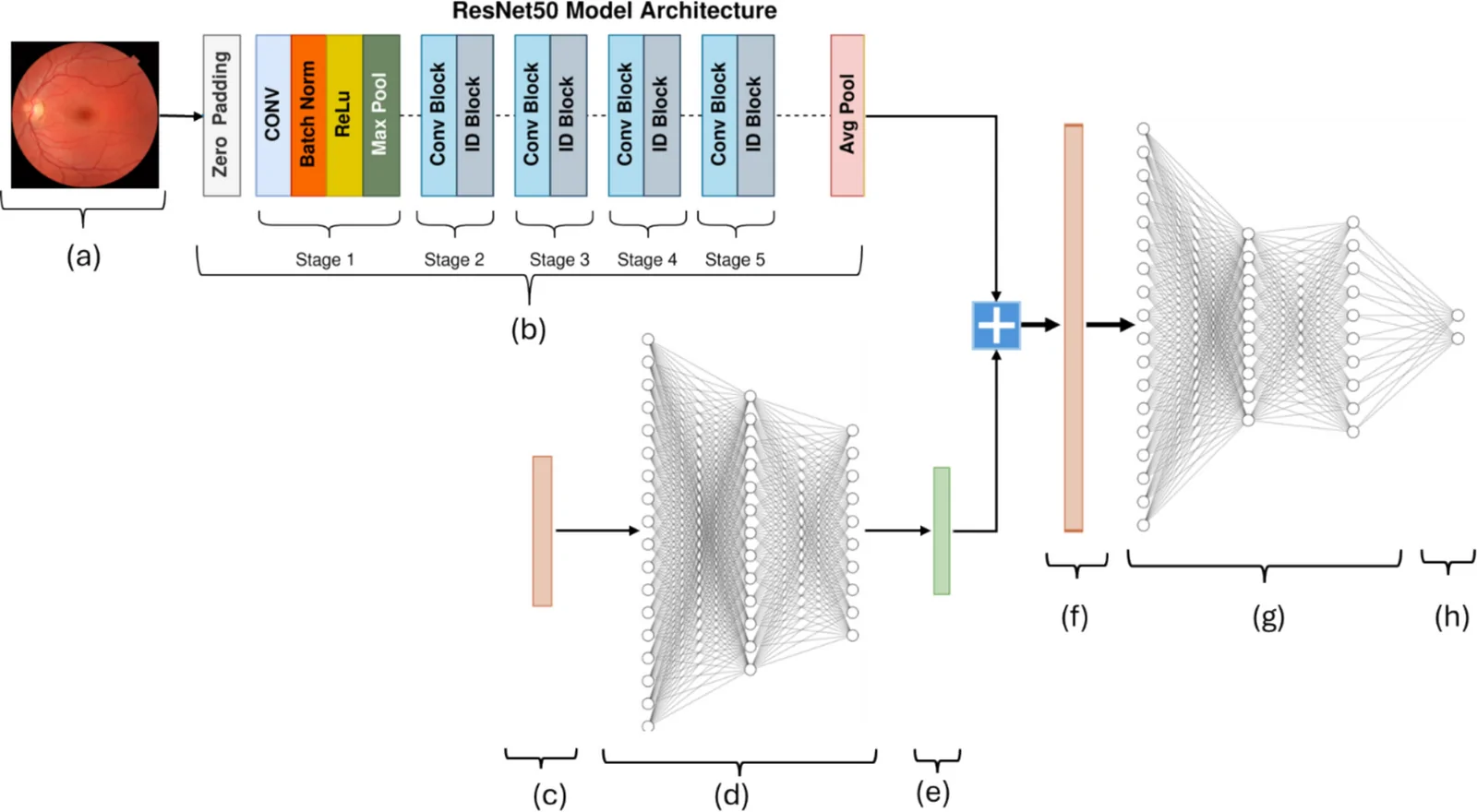
The applied architecture (Topo-RetNet) has two inputs: (**a**) the raw fundus image and (**c**) its corresponding feature vector, which combines topological, LBP, and Gabor features extracted from the same image. The top row shows the CNN-based feature extraction pipeline, where the fundus image is first processed by a previously trained model, which is then fine-tuned on the current dataset. Simultaneously, in the bottom row, (**d**) a feed-forward neural network processes the extracted topological, LBP, and Gabor features to effectively integrate them into the model. The outputs from Avg Pool (CNN-extracted features) and (**e**) (processed features) are then concatenated (+) to form an aggregated feature representation (**f**). Finally, this combined representation is processed into another feed-forward network (**g**) to produce the final classification of the disease pattern (**h**)

Before input to the ResNet50 model, the images were resized to (256, 256) for each color channel. The ResNet50 model was used as a feature extractor, with its pre-trained weights kept fixed. This helps retain valuable learned patterns from ImageNet, reducing the need for training from scratch. The extracted feature map is then processed by a fully connected layer with 64 neurons, with ReLU (Rectified Linear Unit) activation, which introduces nonlinearity and helps the model learn complex patterns in the data. In parallel, topological features were extracted using persistent homology and processed through a separate Multi-Layer Perceptron (MLP). The MLP consists of three layers with 1600, 256, and 128 neurons, utilizing ReLU activation to enhance learning. The outputs from both the CNN and MLP models were then concatenated to create a combined feature representation. These combined features pass through additional fully connected layers with 256 and 128 units, using ReLU activation for better learning. Finally, the classification uses a dense output layer with 5 neurons and SoftMax activation, assigning probabilities to different disease categories. Later, we used the Adam optimizer for parameter tuning and trained the image data using an 80/20 data split. The flowchart of our model is shown in Fig. 6.

### Datasets

To compare the performance of our proposed topological machine learning model and topological deep learning models, we used three open-source datasets for screening Glaucoma, Diabetic Retinopathy (DR), and Age-related Macular Degeneration (AMD). A summary of the key characteristics of these datasets is provided in Table 1.

**Table 1:** Datasets used in this study

Dataset	Disease	Total (N)	Disease	Non-disease
Aptos 2019	Diabetic retinopathy	3662	1805	1857
ORIGA	Glucoma	650	482	168
IChallenge-AMD	Age-related macular degeneration	400	311	89

IChallenge-AMD dataset [26] was designed for the Automatic Detection challenge on Age-related Macular degeneration (ADAM Challenge). The dataset contains 800 retinal fundus images, but only 400 images were labeled. These labeled images were divided into 311 non-AMD and 89 AMD cases. Moreover, the ORIGA dataset (Online Retinal Images for Glaucoma Analysis) [27] is a publicly available dataset designed for glaucoma detection and analysis research. It contains 650 retinal fundus images, with 168 labeled as glaucomatous and the remaining as healthy. Lastly, the APTOS 2019 [28] dataset was introduced as part of the Asia Pacific Tele-Ophthalmology Society (APTOS) Blindness Detection challenge. It is a large-scale dataset used to detect diabetic retinopathy from fundus images. The dataset contains 3,662 high-resolution images, labeled with five levels of diabetic retinopathy severity: 0 (no DR) with 1,805 images, 1 (mild) with 370 images, 2 (moderate) with 999 images, 3 (severe) with 193 images, and 4 (proliferative DR) with 295 images. Medical experts categorize each image based on the presence and severity of lesions. In the binary classification scenario, class 0 represents the normal group, while classes 1–4 comprise the diabetic retinopathy (DR) group, resulting in a class distribution of 1,805 normal images and 1,857 DR images.

## Results

This section evaluates the topological deep learning model (Topo-RetNet) for binary and multi-class classification of DR, glaucoma, and AMD. The performance of the models was compared with several state-of-the-art methods, with metrics including accuracy, precision, recall, F1-score, and AUC reported.

Table 2 presents a performance comparison of various models on the APTOS 2019 dataset for binary classification of DR. The methods evaluated include SCL, ConvNet, DRISTI, Composite DNN, DenseNet121, LBCNN, CNN-Depth Three, and the proposed Topo-RetNet model. The Topo-LBP-G-ML model achieves the highest performance across metrics such as precision, recall, F1-score, accuracy, and AUC, with an impressive AUC of 100 in the 80/20 split and 99.15 in the 80/20 split. The Topo-RetNet model also demonstrates competitive performance, especially in precision and recall, achieving 98.18% recall and an AUC of 100% on the 80/20 split.

**Table 2:** Results for binary disease classification on APTOS 2019

Method	Nor/Abn	Train/Test	Class	Precision	Recall	F1-Score	Accuracy	AUC
SCL [29]	1805/1857	85/15	2	98.17	98.36	98.37	98.36	98.5
ConvNet [30]	1805/1857	80/20	2	-	-	-	97.41	-
DRISTI [31]	1805/1857	80/20	2	-	-	-	97.05	-
Composite DNN [32]	1805/1857	80/20	2	98	98	98	97.82	98
DenseNet121 [33]	1805/1857	85/15	2	86	87	86	94.44	-
LBCNN [34]	1805/1857	80/20	2	-	-	-	96.58	98.71
CNN-Depth Three [35]	1805/1857	-	2	-	-	-	94	-
Topo-Net [15]	1805/1857	85/15	2	98.7	98.7	-	98.7	98.9
Topo-ML [15]	1805/1857	80/20	2	95.5	94	-	94.5	97.9
Topo-LBP-G-ML [36]	1805/1857	80/20	2	97.07	98.12	97.59	97.54	99.15
Topo-RetNet	1805/1857	80/20	2	98.53	98.18	98.35	98.36	100

Table 3 illustrates the performance of various models on the APTOS 2019 dataset in a multi-class classification setting with five DR classes. The dataset was evaluated using different methods, including SCL, DRISTI, DNN, Topo-Net, and Topo-LBP-G-ML. Among the models, the proposed Topo-RetNet achieved the highest accuracy (84.91%) and AUC (96) in the 80/20 split, outperforming other methods. Similarly, Topo-RetML also showed competitive performance, with 80.35% accuracy and an AUC of 92.41% on the 80/20 split. Compared with existing methods, including SCL and DNN, the Topo-RetNet presented in this study outperforms others, particularly in precision and recall, thereby solidifying its effectiveness for multi-class classification in DR detection.

**Table 3:** Results for multi-class disease classification on APTOS 2019

Method	Nor/Abn	Train/Test	Class	Precision	Recall	Accuracy	AUC
SCL [29]	1805/1857	85/15	5	73.84	70.51	84.36	93.82
DRISTI [31]	1805/1857	80/20	5	-	-	75.5	-
DNN [32]	1805/1857	80/20	5	82	83	82.54	79
Topo-Net [15]	1805/1857	85/15	5	82.2	78.2	80	95.5
Topo-ML [15]	1805/1857	80/20	5	73.5	75.6	75.6	88.3
Topo-LBP-G-ML [36]	1805/1857	80/20	5	79.16	80.35	80.35	92.41
Topo-RetNet	1805/1857	80/20	5	84.36	84.91	84.91	96

**Fig. 7:**
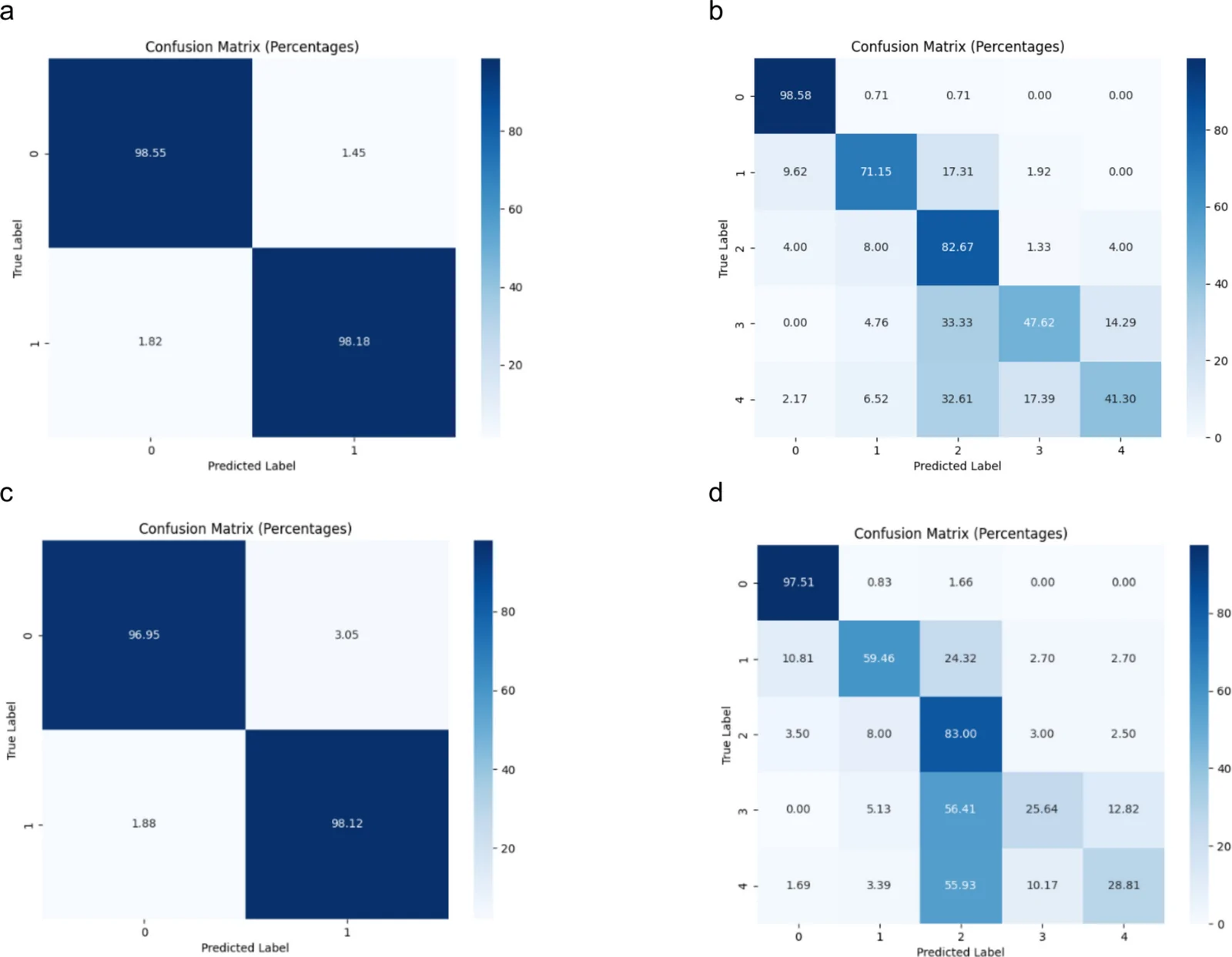
Confusion matrices for the APTOS 2019 dataset. **a** Binary classification using topological deep learning. **b** Multi-class classification using topological deep learning. **c** Binary classification using Topo-MLNet. **d** Multi-class classification using Topo-LBP-G-ML

To further illustrate the classification performance of the models, we present the confusion matrices (see Fig. 7) for both the binary and multi-class settings of the APTOS 2019 dataset. These confusion matrices provide a detailed breakdown of the true positive, false positive, true negative, and false negative rates, offering a clearer understanding of the model’s behavior in classifying normal versus abnormal cases in the binary setting, as well as its ability to distinguish between the five classes in the multi-class setting. By examining these matrices, we can observe the models’ strengths in correctly identifying cases and potential areas of misclassification, providing valuable insights into the proposed approaches’ overall classification accuracy and robustness.

**Fig. 8:**
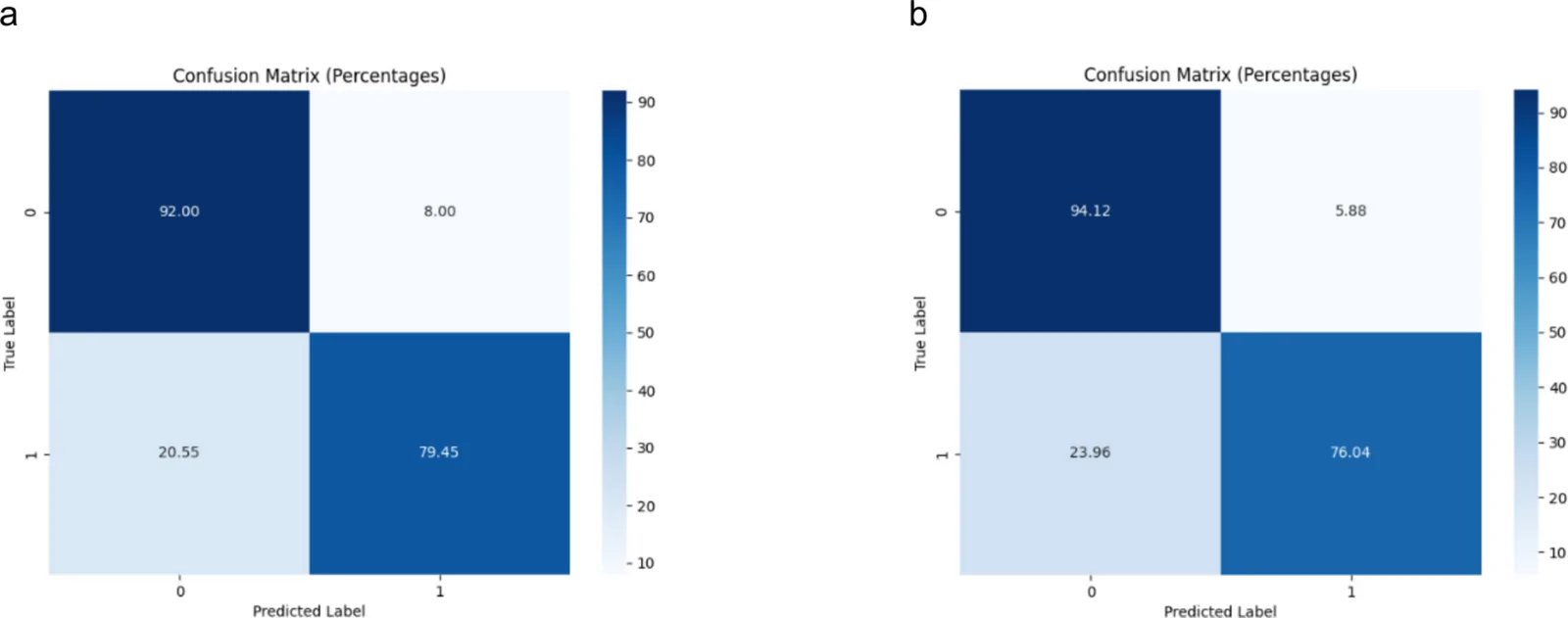
Confusion matrices for the ORIGA dataset. **a** Binary classification using Topo-RetNet. **b** Binary classification using Topo-LBP-G-ML

Table 4 presents a performance comparison of various models on the ORIGA dataset for binary glaucoma classification. Among the models, the pretrained ODGNET achieved the highest sensitivity (94.75%) and accuracy (95.75%), with an AUC of 97.85. Other models, such as En-ConvNet and the 18-layer CNN, demonstrated lower sensitivity (72.2% and 58.06%, respectively) and lower overall accuracy (79.6% and 78.32%, respectively). Our proposed topological model, Topo-RetNet, showed competitive performance. Topo-RetNet achieved a sensitivity of 79.45%, specificity of 92%, accuracy of 82.65%, and AUC of 85.73%. Topo-LBP-G-ML, on the other hand, demonstrated a sensitivity of 76.04%, specificity of 94.12%, accuracy of 80.77%, and an AUC of 85.08. These results indicate that our models achieve strong performance, particularly in terms of accuracy and AUC, outperforming many other approaches in glaucoma diagnosis. The confusion matrices (see Fig. 8) further illustrate the classification performance of the proposed models. In (a), the Topo-RetNet model correctly classified 79.45% of abnormal cases (label 1) and 92% of normal cases (label 0), with a misclassification rate of 20.55% for abnormal cases. In (b), the Topo-LBP-G-ML model correctly classified 76.04% of abnormal cases and 94.12% of normal cases, with 23.96% of abnormal cases misclassified. These matrices highlight the robustness of the proposed topological models for binary classification, with Topo-RetNet showing slightly better sensitivity and specificity than Topo-LBP-G-ML.

**Table 4:** Results for binary disease classification on ORIGA

Method	Nor/Abn	Train/Test	Class	Sensitivity	Specificity	Accuracy	AUC
ODGNET [37]	482/168	Pre-trained	2	94.75	94.90	95.75	97.85
En-ConvNet [38]	482/168	Pre-trained	2	72.2	84.8	79.6	-
18–layer CNN [39]	482/168	70/30	2	58.06	92.44	78.32	-
EAMNet [40]	482/168	tenfold	2	-	-		88
Topo-Net [15]	482/168	85/15	2	84.6	95.8	82.3	83
Topo-ML [15]	482/168	80/20	2	50	92.7	81.5	77.3
Topo-LBP-G-ML [36]	482/168	80:20	2	76.04	94.12	80.77	85.08
Topo-RetNet	482/168	85/15	2	79.45	92	82.65	85.73

**Fig. 9:**
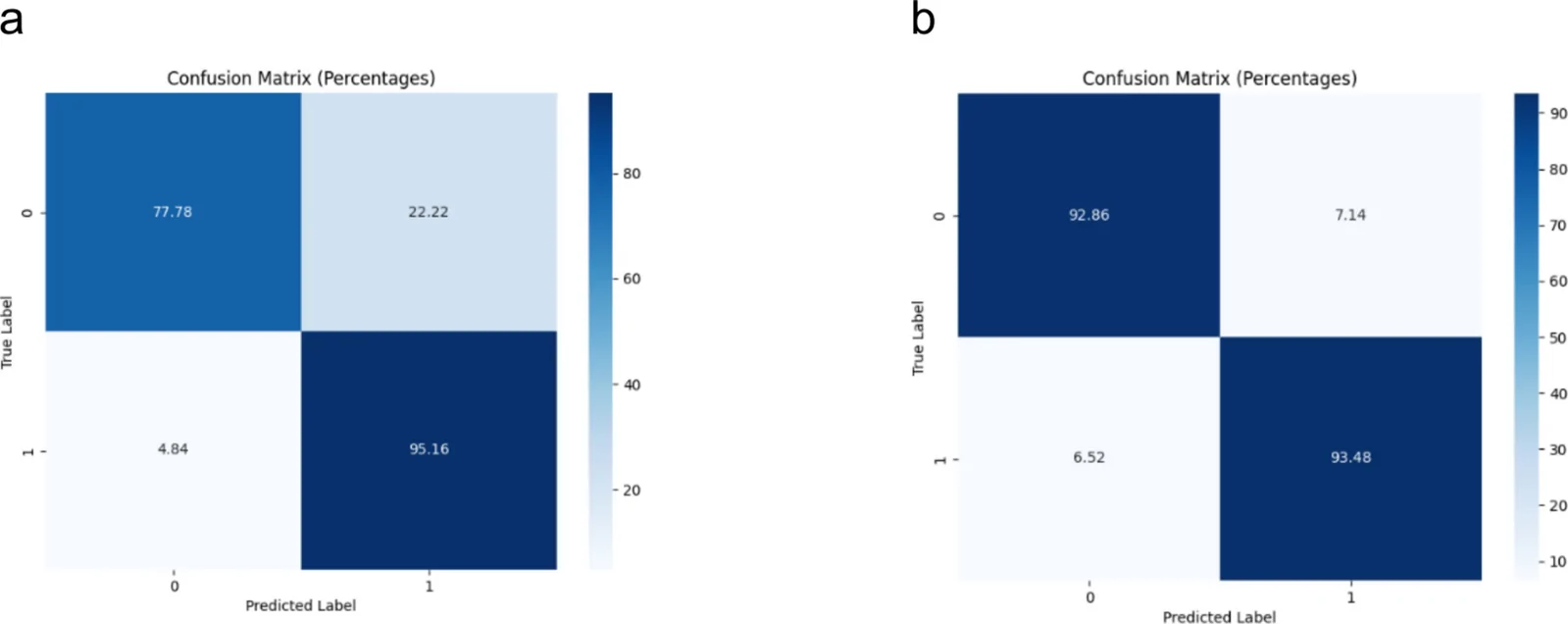
Confusion matrices for the ICHALLENGE-AMD dataset. **a** Binary classification using Topo-RetNet. **b** Binary classification using Topo-LBP-G-M

In Table 5, we evaluated the performance of several models for the binary classification of AMD using the ICHALLENGE-AMD dataset. Pre-existing methods such as Rotation and DCNN achieved accuracies of 87.6% and 91.7%, with AUCs of 77.2% and 88%, respectively. Rotation had a sensitivity of 77.2%, while DCNN achieved 76%. Our proposed models, Topo-RetNet and Topo-LBP-G-ML, exhibit superior performance. The Topo-RetNet model achieved a sensitivity of 93.48%, precision of 97.73%, accuracy of 93.33%, and an AUC of 93.17. Similarly, the Topo-LBP-G-ML model yielded a sensitivity of 91.25%, precision of 93.65%, accuracy of 91.25%, and an AUC of 92.04. These results indicate that our models outperform the baseline approaches in sensitivity and overall classification performance. For the Topo-RetNet model, the confusion matrix shows that 95.16% of abnormal cases (label 1) were correctly classified, and 77.78% of normal cases (label 0) were correctly classified. However, 22.22% of normal cases were misclassified as abnormal, while only 4.84% of abnormal cases were misclassified as normal. The confusion matrix (see Fig. 9) for the Topo-RetML model shows that 93.48% of abnormal cases were correctly classified, while 92.86% of normal cases were correctly identified. The model misclassified 6.52% of abnormal cases and 7.14% of standard cases. These results show that both models perform robustly, with Topo-RetNet slightly outperforming Topo-RetML in precision and sensitivity while maintaining high accuracy and AUC.

**Table 5:** Results for binary disease classification on ICHALLENGE-AMD

Method	Nor/Abn	Train/Test	Class	Sensitivity	Precision	Accuracy	AUC
Rotation [41]	311/89	fivefold cv	2	77.2	84.5	87.6	77.2
DCNN [42]	311/89	tenfold cv	2	76	84.9	91.7	88
Topo-Net [15]	311/89	85/15	2	91.3	92.4	93.8	93.6
Topo-ML [15]	311/89	80/20	2	57.1	88.9	91.3	90.6
Topo-LBP-G-ML [36]	311/89	80/20	2	91.25	93.65	91.25	92.04
Topo-RetNet	311/89	85/15	2	93.48	97.73	93.33	93.17

To evaluate redundancy among feature groups, we calculated the average pairwise Pearson correlations for TDA, LBP, and Gabor features across datasets. This suggests that the feature groups capture largely complementary information, as the results consistently demonstrated low average absolute correlations. To be more precise, the average correlations for the APTOS 2019 dataset were TDA-LBP = 0.153, TDA-Gabor = 0.074, and LBP-Gabor = 0.079. In the same vein, the ORIGA dataset yielded the following values: TDA-LBP = 0.167, TDA-Gabor = 0.082, and LBP-Gabor = 0.091. The average correlations for the IChallenge-AMD dataset were TDA-LBP = 0.148, TDA-Gabor = 0.069, and LBP-Gabor = 0.085. These consistently low inter-group correlations across datasets indicate that topological, local texture, and frequency-based features provide complementary, non-redundant information that is advantageous for classification.

All analyses were performed on a workstation equipped with an NVIDIA GeForce RTX 3070 GPU (8 GB VRAM), Intel Core i7 processor, and 32 GB RAM, running Windows 11 (64-bit). Each CNN-based hybrid model (TDA + CNN) was trained in 3.4 min. Traditional machine-learning models using only TDA features (e.g., XGBoost, Random Forest) trained in under ten seconds. Overall, the computational requirements were modest, and all experiments were completed efficiently on a single consumer-grade GPU without requiring high-performance computing resources.

## Ablation Study

To systematically assess the contribution of different feature extractors and color channels, an ablation study was conducted on the APTOS 2019 dataset under the multi-class classification setting (Table 6). The objective was to quantify how individual and combined feature representations affect classification performance when using the XGBoost classifier. The analysis focused on three handcrafted feature types—Topological Data Analysis (TDA), Local Binary Pattern (LBP), and Gabor filters—and their combinations under different color channel settings (RGB, RGB + HSV).

**Table 6:** Results of the ablation study for multi-class disease classification on the APTOS 2019 dataset

Method	Precision	Recall	Accuracy	AUC
Extractor: TDA Channels: RGB Classifier: XGBoost	72.2	75.4	75.4	88.7
Extractor: LBP Channels: RGB Classifier: XGBoost	76.4	78.6	78.6	91.9
Extractor: GABOR Channels: RGB Classifier: XGBoost	74.4	77.2	77.2	90.1
Extractor: TDA + LBP Channels: RGB Classifier: XGBoost	74.6	77.1	77.1	91.6
Extractor: TDA + GABOR Channels: RGB Classifier: XGBoost	73.9	76.7	76.7	90.3
Extractor: TDA + LBP + GABOR Channels: RGB Classifier: XGBoost	74	77.2	77.2	91.7
Extractor: TDA + LBP + GABOR Channels: RGB + HSV Classifier: XGBoost	79.16	80.35	80.35	92.41

When evaluated independently, LBP features yielded the best individual performance (accuracy = 78.6, AUC = 91.9), surpassing Gabor (accuracy = 77.2, AUC = 90.1) and TDA (accuracy = 75.4, AUC = 88.7). This indicates that fine-grained LBP texture descriptors capture highly discriminative local intensity variations, particularly relevant to retinal lesion patterns such as microaneurysms and exudates. In contrast, the TDA features captured higher-level structural and connectivity information, which, while less directly discriminative alone, provided complementary global insights when combined with texture features.

Combining multiple feature types yielded consistent performance improvements, underscoring their complementary nature. The TDA + LBP and TDA + Gabor combinations improved both precision and recall relative to single extractors, confirming that topological invariants enhance the stability and robustness of texture-based features. The integration of all three—TDA + LBP + Gabor—achieved an accuracy of 77.2 and an AUC of 91.7, demonstrating synergistic benefits from combining topology, texture, and frequency information.

Furthermore, incorporating color information from both the RGB and HSV channels yielded the greatest improvement. The TDA + LBP + Gabor (RGB + HSV) configuration achieved the highest overall performance (precision = 79.16, recall = 80.35, accuracy = 80.35, AUC = 92.41). This enhancement reflects the additional discriminative power obtained from the hue and saturation dimensions in the HSV color space, which capture chromatic and illumination variations more effectively than RGB alone.

Overall, these results reveal that LBP and Gabor features contribute most directly to discriminative performance through fine-texture representation. In contrast, TDA features enhance model robustness and generalization by encoding structural relationships. Their fusion—particularly when combined with multi-channel color information—provides the optimal representation. This analysis supports the hybrid design of the Topo-RetML and Topo-RetNet frameworks, which jointly leverage topological and textural features to achieve superior multi-class disease classification performance.

## Discussion

In this study, we proposed two novel models, the topological machine learning model and the topological deep learning model, that integrate topological features from different color channels with deep learning to enhance the classification of DR, Glaucoma, and AMD using fundus images. Our results demonstrate the robustness and accuracy of these models in both binary and multi-class classification tasks, outperforming several state-of-the-art methods.

One of our approach’s key strengths is combining topological features from different color channels with deep learning features. Topological data with varying color channels enabled us to extract topological features from various patterns, such as connected components and loops, crucial for capturing global structures in retinal images that are often missed by pixel-level methods. By integrating these features with the detailed image representations learned by neural networks, our models can leverage complementary information, thereby improving classification performance across multiple datasets. This highlights the value of topological features and different color channels to extract essential features in medical image analysis, particularly in scenarios where subtle structural differences in retinal images are critical for early diagnosis.

In binary classification for DR detection using the APTOS 2019 dataset, our topological machine learning model achieved the highest AUC in the 85/15 train-test split, demonstrating its superior ability to distinguish between normal and abnormal cases. Similarly, the topological deep learning model performed well, with high precision and recall, indicating that both models effectively handle imbalanced data and reduce false positives and negatives. For multi-class DR classification, the proposed deep learning model outperformed other methods, achieving an accuracy of 84.91% and an AUC of 96%, identifying its robustness in distinguishing between different severity levels of DR. When applied to the ORIGA dataset for glaucoma detection, our models also demonstrated competitive performance, with topological deep learning achieving an accuracy of 82.65% and an AUC of 85.73%. While the pre-trained ODGNET model outperformed our approach in sensitivity and accuracy, our models, particularly the deep learning model, provided an alternative by capturing topological features complementary to those extracted by pre-trained networks. The confusion matrices’ results further supported our models’ effectiveness, as they correctly classified a large proportion of both normal and abnormal cases with relatively low misclassification rates. For AMD detection using the ICHALLENGE-AMD dataset, the proposed deep learning model with topological features showed superior performance, with an accuracy of 93.33% and an AUC of 93.17%. The confusion matrix showed that the model achieved high precision in correctly classifying abnormal cases. Topo-RetML also performed well, achieving 91.25% accuracy, though Topo-RetNet slightly outperformed it in terms of sensitivity and specificity.

These results suggest that integrating topological features with different color channels with neural network-based architectures is a promising approach for pattern recognition and retinal disease classification, especially for tasks that require global and local feature extraction. The robustness of our models across different datasets and classification tasks further validates their generalizability and potential for broader clinical applications. However, our study has some limitations. First, while our models performed well across different datasets, they were tested only on publicly available data, which may not fully reflect the variability observed in real-world clinical settings. Additionally, although combining topological features and deep learning features proved beneficial, the computational cost of generating topological features and training deep learning models can be high. Future work could explore optimizing the feature extraction process to reduce computational overhead while maintaining performance. Overall, our proposed models, topological machine learning and topological deep learning methods, demonstrate significant improvements over existing methods in pattern recognition and retinal disease classification. Combining features across topology and color channels is a powerful tool for enhancing diagnostic accuracy, potentially leading to earlier detection and improved patient outcomes in clinical practice.

## Data Availability

The datasets used in this study are publicly available.
